# Recurrent Intracerebral Haematomas Due to Amyloid Angyopathy after Lyodura Transplantation in Childhood

**DOI:** 10.3390/neurolint16020023

**Published:** 2024-03-04

**Authors:** Maša Fabjan, Ana Jurečič, Miha Jerala, Janja Pretnar Oblak, Senta Frol

**Affiliations:** 1 Department of Vascular Neurology, University Medical Center Ljubljana, Zaloška 2, 1000 Ljubljana, Slovenia; hafner.masa@gmail.com (M.F.); ana.jurecic22@gmail.com (A.J.); janja.pretnar@kclj.si (J.P.O.); 2 Institute of Pathology, University of Ljubljana, 1000 Ljubljana, Slovenia; miha.jerala@mf.uni-lj.si; 3 Faculty of Medicine, University of Ljubljana, Zaloška 2, 1000 Ljubljana, Slovenia

**Keywords:** iatrogenic cerebral amyloid angiopathy, LYODURA, recurrent intracerebral haematomas

## Abstract

The number of published cases of presumed iatrogenic cerebral amyloid angiopathy (iCAA) due to the transmission of amyloid β during neurosurgery is slowly rising. One of the potential ways of transmission is through a cadaveric dura mater graft (LYODURA) exposure during neurosurgery. This is a case of a 46-year-old female patient with no chronic conditions who presented with recurrent intracerebral haemorrhages (ICHs) without underlying vessel pathology. Four decades prior, the patient had a neurosurgical procedure with documented LYODURA transplantation. Brain biopsy confirmed CAA. This is a rare case of histologically proven iCAA after a documented LYODURA transplantation in childhood. Our case and already published iCAA cases emphasize the need for considering neurosurgery procedure history as important data in patients who present with ICH possibly related to CAA.

## 1. Introduction

Cerebral amyloid angiopathy (CAA) is a disease caused by amyloid β (Aβ) deposition in the vascular walls [1,2]. It results in the loss of smooth muscle in the vessel wall, leading to aneurysms, micro bleeding and vascular occlusions [1,2]. CAA can present as intracerebral haemorrhage (ICH)—mostly lobar, sulcal subarachnoid haemorrhage, seizures, transient focal neurological episodes and cognitive decline [2,3]. CAA is mostly a sporadic disease of the elderly. However, occasionally, it does occur in younger patients due to specific genetic mutations of Aβ such as APP mutations or duplications and PSEN1 or PSEN2 mutations [4]; it can also be associated with inflammation, significant traumatic brain injury (TBI) [5] and neurosurgery in childhood [3,4]. A review of the literature suggests that a history of significant traumatic brain injury (TBI), predominantly in young male patients, may be associated with CAA [4,5].

Even today, a definite diagnosis of CAA is based on the pathological findings. In vivo radiological criteria enable us to establish probable and possible CAA. Edinburgh criteria with computed tomography (CT) head scans [6] and Boston criteria with magnetic resonance imaging (MRI) head scans [7] are used. Reduced levels of Aβ, particularly Aβ-42 and ratio Aβ-42/Aβ-40, high values of p-Tau and Tau protein in the cerebrospinal (CSF) fluid, and amyloid positron emission tomography (PET) imaging have also been of a relevant diagnostic value [3,4].

As already mentioned in the last few years, iatrogenic CAA (iCAA), which has similarities to prion diseases, has been recognized [4,8]. The transmission of Aβ during neurosurgery with the use of a cadaveric material (like cadaveric dura mater or dura of uncertain type, human cadaveric pituitary-derived growth hormone and possibly even surgical instrument) in the central nervous system (CNS) can occur in childhood. This may result in an early onset iCAA. Patients with iCAA present decades after CNS (brain or spinal cord) neurosurgery exposure with the same clinical presentation and neuroradiological features as patients with CAA [3,4]. Pikija et al. [4] recently published the largest review of case reports and case series up to date that link exposure to cadaveric material used during childhood neurosurgery procedures to middle-aged or young patients with iCAA, regardless of the type or location of exposure [4]. ICH was present in 82% of patients in their cohort and 11% of patients presented with seizures as the first presentation symptom [4]. Patients with iCAA are usually middle-aged and, therefore, significantly younger compared to patients with a typical sporadic CAA [3,4]. The knowledge of prognosis, outcome and management of iCAA is still scarce. Pikija et al. [4] described only eight cases of cadaveric dura mater graft (LYODURA) (B.Braun, Melsungen, Germany) transplantation in childhood and six cases of uncertain dura-type placement during neurosurgery in childhood with CAA development several decades later [4].

With the discovery of the variant Creutzfeldt–Jakob disease, the awareness of possible prion disease seeding increased. Hence, the use of cadaveric material was omitted, and the sterilization of surgical instruments improved. LYODURA was, therefore, used primarily between the years 1970 and 1995 [4]. As a consequence, the cases of iCAA should become rarer or possibly not appear at all. It is, therefore, imperative to recognize and analyse them now. Recently, the Queen Square diagnostic criteria (QSC) for iCAA [9] were published, which aid in establishing iCAA as probable or possible.

Here, we describe a new case of a middle-aged patient in whom LYODURA was placed during neurosurgery in childhood and who developed CAA with multiple ICHs several decades later.

## 2. Case Report

A 46-year-old Caucasian right-handed woman without known chronic conditions was admitted to the neurology emergency department at the University Medical Centre (UMC), Ljubljana, due to a sudden transient loss of consciousness, followed by severe headache with vomiting and left-sided hemiplegia. At the age of 9 years (in 1985), she had a neurosurgical removal of a benign tumour of the left temporal lobe, and LYODURA transplant was used for defect closure. Her family history of neurological disorders was insignificant.

**Figure 1:**
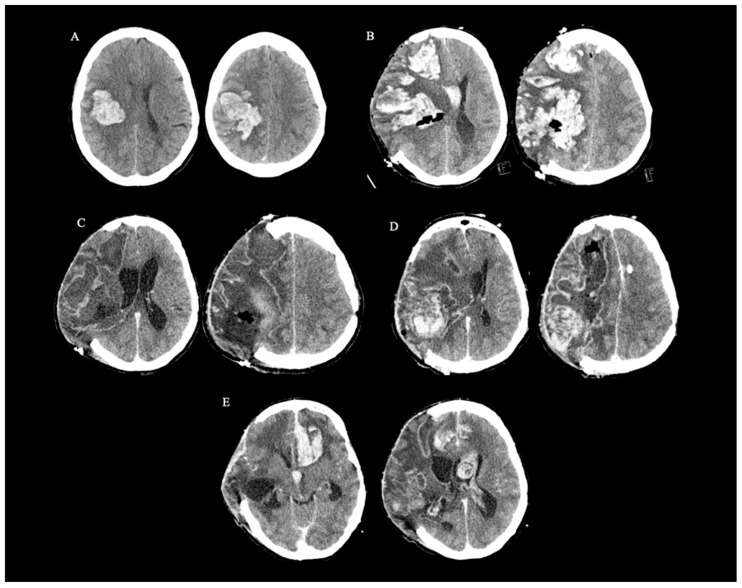
Head computed tomography (CT) scans of the patient at different time intervals. (**A**) at admission showing a 6 × 3 cm cortical—subcortical intracerebral haematoma (ICH) with surrounding cerebral oedema in the right temporoparietal lobe with subfalcine herniation. (**B**) Postoperatively showing a massive ICH in the right frontotemporoparietal region with haematocephalus, cerebral oedema and subfalcine herniation. (**C**) A month after admission (head CT scan with contrast) showing a 15 × 8 × 9 cm intracranial abscess in the right hemisphere. (**D**) Postoperatively (head CT with contrast) showing residual abscess with a recurrent ICH in the right parietal region with subfalcine, uncal and descendent transtentorial herniation. (**E**) Two months after admission showing a new ICH in the left frontal region and blood in the left lateral ventricle.

Her vital signs at admission were normal, she was awake (Glasgow Coma Scale score 15), oriented, and without speech impairment. Meningeal signs were negative, and she was afebrile. She had a right head and eyes deviation, left nasolabial fold asymmetry, left-sided hemisensory loss and left-sided flaccid hemiplegia, with a left plantar response in extension (National Stroke Scale score 15, modified Rankin score 5). An initial head CT scan showed a large ICH in the right temporoparietal lobe (Figure 1A). CT angiography (aortocervical and intracranial) did not reveal any underlying vessel pathology. The basic laboratory tests including blood cell count, inflammatory parameters, blood coagulation (international normalized ratio (INR) and activated partial thromboplastin time (APTT) were normal.

An emergency surgical removal of ICH was performed, but, intraoperatively, fulminant cerebral oedema developed. She received antioedema intravenous treatment, a decompressive craniotomy was performed and an intracranial pressure (ICP) monitor was inserted. Even though the biopsy was planned, it was not performed because of the complications and fragile brain tissue. She was transferred to the neurocritical care unit. A postoperative CT scan showed a recurrent massive ICH in the right frontotemporoparietal region (Figure 1B). In addition, CT venography was normal, and coagulation tests (INR, APTT, thrombin time (TT)) were unremarkable. Autoimmune diseases (lupus anticoagulant, antiphospholipid antibodies, ANCA) were excluded.

During the next two weeks, despite several pharmacological and non-pharmacological treatments, ICP levels remained elevated up to 55 cm H_2_O. Multiple head CT scans were performed and showed different stages of ICH reabsorption with massive cerebral oedema resulting in subfalcine and transtentorial herniation. A month after admission, a head CT scan with contrast showed a massive intracranial abscess in the right hemisphere (Figure 1C) with ventriculitis; therefore, a double intravenous antibiotic treatment was initiated. Although the abscess was surgically removed, the control head CT scan showed a recurrent ICH with residual abscess in the right parietal region (Figure 1D). After oedema stabilized, sedation was discontinued, but the patient did not regain consciousness. She developed frequent paroxysms of autonomic instability with dystonia. She was successfully extubated but remained dysphagic with impaired cough reflex. A week after sedation discontinuation, a control head CT scan showed a new ICH in the left frontal region and haematocephalus (Figure 1E). Due to recurrent ICHs, possible CAA and complications during previous surgical procedures, palliative care was implemented. Additional head CT scans in the following weeks showed multiple new ICHs in the left and right hemispheres. She died due to multiorgan failure three months after admission. CSF examination was not performed due to repeated ICHs. PET for amyloid was also not performed. Exome sequencing performed revealed no pathogenic variants in the genes associated with amyloid angiopathy.

**Figure 2:**
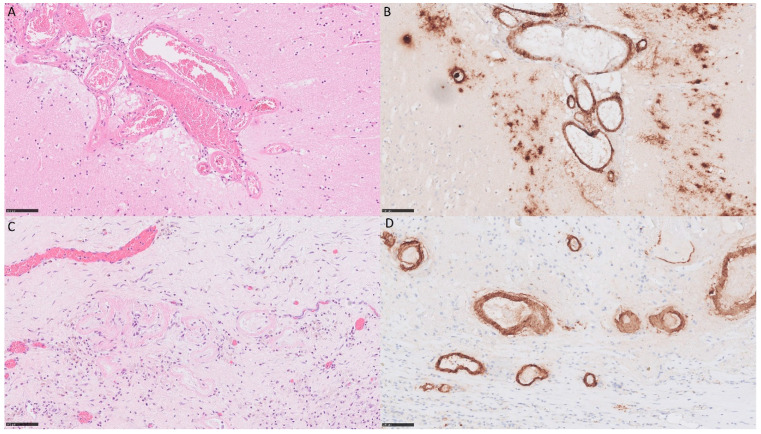
Representative sections of brain biopsy. Haematoxylin and eosin sections of the (**A**) occipital lobe and (**C**) dura showing thickened blood vessels with eosinophilic amorphous deposits. Amyloid β immunohistochemically staining of (**B**) the occipital lobe and (**D**) dura show extensive vascular amyloid β deposition as well as perivascular plaques in the cortex. (200× magnification, scale bar = 100 µm).

The brain autopsy was performed. The brain was examined after fixation in 10% buffered formalin. The brain weighted 2055 g, with flattened gyri and signs of tonsillar and tentorial herniation. Multiple parenchymal haemorrhages were evident bilaterally in the frontal, temporal and parietal lobes as well as in the basal ganglia and part of the right occipital lobe. The brainstem showed secondary haemorrhages after herniation. The brain was extensively sampled for histological examination. Since the exact location of the dural graft could not be identified, a representative sample of dura was taken. Microscopically, in the cortex and basal ganglia, leptomeningeal and parenchymal blood vessel walls were thickened, with amorphic eosinophilic deposits, which were Congo red-positive and showed apple-green birefringence under polarization. Immunohistochemically staining for Aβ showed extensive positivity in the affected blood vessels as well as numerous diffuse and rare neuritic plaques in the temporal and occipital cortex (Figure 2). The sampled dura contained rare blood vessels with Aβ deposits. The arteries of the circle of Willis were free of Aβ.

## 3. Discussion

In this manuscript, we present a middle-aged patient with LYODURA transplantation in childhood who developed a histologically proven CAA four decades later. Regarding the QSC, our patient met the criteria for probable iCAA [9].

Due to the rarity and novelty of the disease, current real-world data on iCAA is scarce, although the number of cases reported in the literature is rising slowly [4,10,11,12,13,14,15,16,17,18,19,20,21,22,23,24,25,26,27,28]. The largest multinational cohort of iCAA patients recently published by Pikija et al. [4] included only 27 patients. Our patient presented with neurological deficits associated with lobar ICH seen on admission, head CT scan and transient loss of consciousness most probably due to epileptic seizure. Clinical presentation and neuroimaging findings were, therefore, typical for CAA patients. At the point of ICH onset, our patient was 46 years old, which is compliant with data published by Pikija et al. [4] in which the authors reported that the average age of the first clinical presentation was the late 40 s (median age 49 years) [4], while Banerjee et al. [9] reported the clinical onset of iCAA presentation at the mean age of 38 years [9]. Pikija et al. predicted that a higher age at the disease presentation could be due to increased awareness of iCAA diagnosis, particularly regarding older patients [4]. Nevertheless, it is also worth mentioning that our patient was female, which is not typical regarding the published literature [4,9]. A female gender has been described in only 22% of previous cases [4]. Pikija et al. speculated that it can reflect a male predisposition to significant TBI, which was also the main cause of neurosurgery in the cohort described by Pikija et al. [4,5].

Our patient had a well-documented LYODURA transplantation during a CNS neurosurgery procedure at the age of 9 years, which is in accordance with the mean age of 11 years at exposure to neurosurgery described by Pikija et al. [4], while Banerjee et al. [9] described a slightly earlier relevant exposure at the age of 6 years. Furthermore, our patient suffered from multiple ICHs 37 years after a neurosurgery procedure, which is, again, within the reported mean latency period of 39 years after neurosurgical exposure [4] and 2 years longer as reported by Banerjee et al. [9]. According to the Edinburgh head CT criteria, our patient had a definite CAA, which makes it one of only 20 published cases of probable iCAA.

Physicians should be aware of the iCAA diagnosis, especially if the location of ICH is lobar or even recurrent, all typical causes of ICH are excluded and the patient had a history of CNS neurosurgical procedure in childhood. Exposure to any cadaveric material such as meninges, brain and pituitary-derived hormones could result in iCAA development [9]; therefore, according to the QSC, this criterion is strongly suggestive of iCAA [9]. There are also some reports of insufficiently sterilized neurosurgical instruments and possible iCAA development with possible suggestion that certain proteinaceous particles could survive sterilization procedures [29,30], but as written by Pikija et al. [4], this hypothesis is still very controversial. As the technology and standards for a high safety in CNS neurosurgical procedures are constantly rising, we can speculate that the number of patients with iCAA due to CNS neurosurgery procedures will decline. Nonetheless, it is also worth mentioning that there are also some publications describing patients suffering significant TBI in early childhood without neurosurgical procedures and developing an early onset CAA [5,22,31,32], with the male sex being a predisposing factor.

Clinicians should consider iCAA in patients presenting with ICHs suggestive of CAA aetiology and be more aware of taking patients’ medical history regarding CNS neurosurgical procedures performed in early childhood. Brain biopsy or autopsy should be performed in order to establish a definite diagnosis of CAA. Importantly, even larger multinational registries are mandatory for further exploration of this novel rare disease. Using the QSC as standardized criteria for iCAA will aid clinicians in the wider recognition of the disease.

## 4. Conclusions

We present a rare case with a well-documented cadaveric LYODURA transplant during an early childhood CNS surgical procedure and a consequential development of recurrent ICHs several decades later due to iCAA, confirmed by brain biopsy. It is important to consider the history of neurosurgical procedures in childhood, especially using cadaveric material in young or middle-aged patients with ICH suggesting the iCAA aetiology, and establish histological proof. Larger multinational registries of patients with iCAA are needed for further investigation of this disease.

## Data Availability

All the data generated or analysed during the study are included in this article. Further inquiries can be directed to the corresponding author.

## References

[1] Weber S.A., Patel R.K., Lutsep H.L. (2018). Cerebral amyloid angiopathy: Diagnosis and potential therapies. Expert Rev. Neurother..

[2] Miller-Thomas M.M., Sipe A.L., Benzinger T.L., McConathy J., Connolly S., Schwetye K.E. (2016). Multimodality Review of Amyloid-related Diseases of the Central Nervous System. Radiographics.

[3] Banerjee G., Collinge J., Fox N.C., Lashley T., Mead S., Schott J.M., Werring D.J., Ryan N.S. (2023). Clinical considerations in early-onset cerebral amyloid angiopathy. Brain.

[4] Pikija S., Pretnar-Oblak J., Frol S., Malojcic B., Gattringer T., Rak-Frattner K., Staykov D., Salmaggi A., Milani R., Magdic J. (2023). Iatrogenic cerebral amyloid angiopathy: A multinational case series and individual patient data analysis of the literature. Int. J. Stroke.

[5] Oblak J.P., Jurečič A., Writzl K., Frol S. (2022). Preceding Head Trauma in Four Cases of Sporadic Cerebral Amyloid Angiopathy—Case Report Series. J. Stroke Cerebrovasc. Dis..

[6] Rodrigues M.A., Samarasekera N., Lerpiniere C., Humphreys C., McCarron M.O., White P.M., Nicoll J.A.R., Sudlow C.L.M., Cordonnier C., Wardlaw J.M. (2018). The Edinburgh CT and genetic diagnostic criteria for lobar intracerebral haemorrhage associated with cerebral amyloid angiopathy: Model development and diagnostic test accuracy study. Lancet Neurol..

[7] Greenberg S.M., Charidimou A. (2018). Diagnosis of Cerebral Amyloid Angiopathy: Evolution of the Boston Criteria. Stroke.

[8] Jaunmuktane Z., Mead S., Ellis M., Wadsworth J.D., Nicoll A.J., Kenny J., Launchbury F., Linehan J., Richard-Loendt A., Walker A.S. (2015). Evidence for human transmission of amyloid-β pathology and cerebral amyloid angiopathy. Nature.

[9] Banerjee G., Samra K., Adams M.E., Jaunmuktane Z., Parry-Jones A.R., Grieve J., Toma A.K., Farmer S.F., Sylvester R., Houlden H. (2022). Iatrogenic cerebral amyloid angiopathy: An emerging clinical phenomenon. J. Neurol. Neurosurg. Psychiatry.

[10] Banerjee G., Adams M.E., Jaunmuktane Z., Alistar Lammie G., Turner B., Wani M., Sawhney I.M.S., Mead S., Brandner S., Werring D.J. (2019). Early onset cerebral amyloid angiopathy following childhood exposure to cadaveric dura. Ann. Neurol..

[11] Jaunmuktane Z., Quaegebeur A., Taipa R., Viana-Baptista M., Barbosa R., Koriath C., Sciot R., Mead S., Brandner S. (2018). Evidence of amyloid-β cerebral amyloid angiopathy transmission through neurosurgery. Acta Neuropathol..

[12] Muller C. (2023). Case report of iatrogenic cerebral amyloid angiopathy after exposure to Lyodura: An Australian perspective. Front. Neurosci..

[13] Purrucker J.C., Röcken C., Reuss D. (2023). Iatrogenic cerebral amyloid angiopathy rather than sporadic CAA in younger adults with lobar intracerebral haemorrhage. Amyloid.

[14] Kaushik K., van Etten E.S., Siegerink B., Kappelle L.J., Lemstra A.W., Schreuder F.B.M., Klijn C.J.M., Peul W.C., Terwindt G.M., van Walderveen M.A.A. (2023). Iatrogenic Cerebral Amyloid Angiopathy Post Neurosurgery: Frequency, Clinical Profile, Radiological Features, and Outcome. Stroke.

[15] Lázaro Romero A., Moreno Loscertales C., Marta Moreno E. (2022). Unilateral cerebral amyloid angiopathy after neurointervention. Neurologia (Engl. Ed.).

[16] Kellie J.F., Campbell B.C.V., Watson R., Praeger A.J., Nair G., Murugasu A., Rowe C.C., Masters C.L., Collins S., McLean C. (2022). Amyloid-β (Aβ)-Related Cerebral Amyloid Angiopathy Causing Lobar Hemorrhage Decades After Childhood Neurosurgery. Stroke.

[17] Limpo H., Andrés A., Fortes J., García M.A., Presti A.L. (2021). Early-onset cerebral amyloid angiopathy, a prion-like disease: Case report and literature review. J. Neurosurg. Res. Rev..

[18] Yoshiki K., Hirose G., Kumahashi K., Kohda Y., Ido K., Shioya A., Misaki K., Kasuga K. (2021). Follow-up study of a patient with early onset cerebral amyloid angiopathy following childhood cadaveric dural graft. Acta Neurochir. (Wien).

[19] Giaccone G., Maderna E., Marucci G., Catania M., Erbetta A., Chiapparini L., Indaco A., Caroppo P., Bersano A., Parati E. (2019). Iatrogenic early onset cerebral amyloid angiopathy 30 years after cerebral trauma with neurosurgery: Vascular amyloid deposits are made up of both Aβ40 and Aβ42. Acta Neuropathol. Commun..

[20] Jaunmuktane Z., Banerjee G., Paine S., Parry-Jones A., Rudge P., Grieve J., Toma A.K., Farmer S.F., Mead S., Houlden H. (2021). Alzheimer’s disease neuropathological change three decades after iatrogenic amyloid-β transmission. Acta Neuropathol..

[21] Hervé D., Porché M., Cabrejo L., Guidoux C., Tournier-Lasserve E., Nicolas G., Adle-Biassette H., Plu I., Chabriat H., Duyckaerts C. (2018). Fatal Aβ cerebral amyloid angiopathy 4 decades after a dural graft at the age of 2 years. Acta Neuropathol..

[22] Nakayama Y., Mineharu Y., Arawaka Y., Nishida S., Tsuji H., Miyake H., Yamaguchi M., Minamiguchi S., Takagi Y., Miyamoto S. (2017). Cerebral amyloid angiopathy in a young man with a history of traumatic brain injury: A case report and review of the literature. Acta Neurochir. (Wien).

[23] Caroppo P., Marucci G., Maccagnano E., Gobbo C.L., Bizzozero I., Tiraboschi P., Redaelli V., Catania M., Di Fede G., Caputi L. (2021). Cerebral amyloid angiopathy in a 51-year-old patient with embolization by dura mater extract and surgery for nasopharyngeal angiofibroma at age 17. Amyloid.

[24] Hamaguchi T., Komatsu J., Sakai K., Noguchi-Shinohara M., Aoki S., Ikeuchi T., Yamada M. (2019). Cerebral hemorrhagic stroke associated with cerebral amyloid angiopathy in young adults about 3 decades after neurosurgeries in their infancy. J. Neurol. Sci..

[25] Ehling R., Helbok R., Beer R., Lackner P., Broessner G., Pfausler B., Röckenb C., Aguzzic A., Chemellid A., Schmutzharda E. (2012). Recurrent intracerebral haemorrhage after coitus: A case report of sporadic cerebral amyloid angiopathy in a younger patient. Eur. J. Neurol..

[26] Michiels L., Van Weehaeghe D., Vandenberghe R., Demeestere J., Van Laere K., Lemmens R. (2021). The Role of Amyloid PET in Diagnosing Possible Transmissible Cerebral Amyloid Angiopathy in Young Adults with a History of Neurosurgery: A Case Series. Cerebrovasc. Dis..

[27] Raposo N., Planton M., Siegfried A., Calviere L., Payoux P., Albucher J.F., Viguier A., Delisle M.-B., Uro-Coste E., Chollet F. (2020). Amyloid-β transmission through cardiac surgery using cadaveric dura mater patch. J. Neurol. Neurosurg. Psychiatry.

[28] Tachiyama K., Kajikawa S., Nakamori M., Matsushima H., Imamura E., Wakabajashi S., Urakami K. (2020). Infant critical head injury could be a remote cause of middle-aged cerebral amyloid angiopathy. Res. Sq..

[29] Thomzig A., Wagenführ K., Daus M.L., Joncic M., Schulz-Schaeffer W.J., Thanheiser M., Mielke M., Beekes M. (2014). Decontamination of medical devices from pathological amyloid-β-, tau- and α-synuclein aggregates. Acta Neuropathol. Commun..

[30] Eisele Y.S., Bolmont T., Heikenwalder M., Langer F., Jacobson L.H., Yan Z.X., Roth K., Aguzzi A., Staufenbiel M., Walker L.C. (2009). Induction of cerebral beta-amyloidosis: Intracerebral versus systemic Abeta inoculation. Proc. Natl. Acad. Sci. USA.

[31] Purrucker J.C., Hund E., Ringleb P.A., Hartmann C., Rohde S., Schönland S., Steiner T. (2013). Cerebral amyloid angiopathy—An underdiagnosed entity in younger adults with lobar intracerebral hemorrhage?. Amyloid.

[32] Campbell D.M., Bruins S., Vogel H., Shuer L.M., Wijman C.A. (2008). Intracerebral hemorrhage caused by cerebral amyloid angiopathy in a 53-year-old man. J. Neurol..

